# Multiple wave scattering by submerged obstacles in an infinite channel of finite depth with surface pressure excess

**DOI:** 10.1038/s41598-024-51512-x

**Published:** 2024-01-23

**Authors:** M. S. Abou-Dina, A. F. Ghaleb, N. S. Abdelrahman

**Affiliations:** 1 Department of Mathematics, Faculty of Science, Cairo University, 12613 Giza, Egypt; 2 Department of Mathematics, Faculty of Science, Suez Canal University, Ismailia, Egypt

**Keywords:** Engineering, Mathematics and computing, Physics

## Abstract

The objective is to study the combined effect of an incident wave, a surface pressure excess and a finite number of submerged obstacles, in the phenomenon of power transfer to an infinite fluid layer of finite depth. The incident wave and the surface pressure excess have the same harmonic time dependence, a fact that allows to eliminate time altogether and consider only steady-state solutions. The surface pressure excess simulates the effect of winds blowing above the water surface in oceans. The technique used in a first part of the paper relying upon the use of finite Fourier transform and separation of variables is extended here to this end. The method allows to separate local perturbations from progressive or standing wave. Our formulae yield the exact solution in closed form in the absence of obstacles, and provide a clearer insight into the flow properties, as compared to previous investigations. Applications are given for discontinuous surface pressure functions. We put in evidence solutions with no outgoing waves, for which the energy transmitted by the surface pressure is exhausted in generating a standing wave, together with local perturbations. Two numerical applications without/with obstacles, for a parabolic surface pressure profile, allow to assess the energy transfer from the pressure-obstacles system to the fluid. The results may be of interest in the field of oscillating water columns and, generally, water power converting technology.

## Introduction

In a first part^1^, denoted (*I*), the authors had presented a semi-analytical approximate solution to the problem of multiple scattering of an incident harmonic wave by a finite number of obstacles submerged in an infinite, homogeneous fluid layer under constant (zero) surface pressure. This is an extension of the work by Abou-Dina and Hassan^2^ concerning the flow over a topography. In (*I*), the solution was found within the linear theory using a hybrid method based on the powerful finite Fourier transform technique, the methods of separation of variables, fundamental solutions and boundary collocation. The governing equations and boundary conditions were satisfied exactly, apart from the impermeability conditions on the obstacles satisfied approximately by collocation.

The technique used in (*I*) is successfully extended here, with minor modifications, to yield a solution for the case when an external surface pressure excess of harmonic time dependence is applied to a finite portion of the free surface. This is the inverse problem of what is commonly known in the literature under the general title of wave-power absorption, when the wave energy is converted into other forms of energy. The most common application is the oscillating water columns. Other applications of the variable surface pressure concern Oceanography, particularly during periods of climate variations. For the sake of conciseness, we have made reference here to a limited number of references tightly related to our present interests. An ample list of references may be found in^1^.

The theory of wave generation by a variable surface pressure in the absence of obstacles, was discussed by Stoker^3^, Ch. 4 and Wehausen & Laitone^4^, pp. 592-597, with reference to earlier literature. These authors gave an analytic formula for the two-dimensional velocity potential relying on methods of complex analysis. An asymptotic analysis was carried out in order to discover the behavior of the solution far from the area where the surface pressure is applied. A formula was deduced for the amplitudes of the outgoing waves in both directions. Stoker^3^, Ch. 4 considered certain particular pressure distributions of harmonic time dependence. He noted a part of the solution, other than the outgoing waves, disappearing as the inverse distance from this area. In addition, he pointed out at exceptional frequencies for which the outgoing waves cease to exist, in which case the energy transmitted by the pressure to the fluid manifests itself in generating a standing wave. Such frequencies are related to the width of the support of the function of surface pressure. Miles^5^ studied the flow generated by an oscillating point pressure in a semi-infinite liquid. Other investigations have concentrated on power absorption by immersed obstacles^6,7^. Evans^8^ investigated the energy absorption of a system of uniform oscillatory surface pressure distributions. Sarmento and Falc$\tilde{a}$o^9^ developed a two-dimensional analysis based on^4^ for an oscillatory wave energy device in water of arbitrary constant depth. In the cited references, little attention was devoted to the local perturbations generated in the fluid, especially at possible points of discontinuity of the variable surface pressure, while principally concentrated on the asymptotic behavior of the solutions at large distances from the pressure support. Abou-Dina and Helal^10–13^ investigated nonlinear problems for the influence of submerged obstacles in stratified fluids, and gravity wave generation by an applied variable pressure in an infinite fluid mass of finite depth within the shallow water theory. They reduced this latter problem to a system of integro-differential equations. Gravity waves generated by moving disturbances were investigated by Darmon *et al.*^14^ and by Benzaquen *et al.*^15^. Li and Ellingsen^16,17^ investigated initial-value problems of water waves with a shear current of uniform vorticity beneath the surface in three dimensions, under the effects of gravity and a distributed external pressure disturbance, with numerical results for the concrete cases. Li *et al.*^18^ studied waves and wave-making forces acting on ships travelling on currents which vary as a function of depth. More recent work on wave transmission and reflection in oceans and effect on ship motion may be found in^19^. Moving localized atmospheric surface pressure disturbances traveling at supercritical or subcritical speeds with real situation application were investigated by Liu and Higuera^20^. The effect of obstacles on wave transmission and reflection under shallow water theory was studied by Al Arfaj *et al.*^21^.

The present work uses a slightly modified version of the method presented in (*I*) to treat the problem of mutiple scattering of an incident harmonic wave by obstacles placed in an infinite channel of finite depth. The fluid surface is acted upon by a pressure excess of finite support, having the same time dependence as the incident wave. Unlike other treatments, the proposed solution consists of three distinct parts: (i) Outgoing waves in both directions, (ii) local perturbations dying out far away from the obstacles and the pressure support, expressed by an infinite series of decaying exponentials, (iii) effect of the system of obstacles. In certain cases, the outgoing waves are suppressed and replaced by a standing wave localized in the neighborhood of the area acted upon by the pressure excess. Analytical formulae are provided for the reflection and the transmission coefficients. Our formulae give a deeper insight into the flow characteristics, as compared to previous investigations available in the literature. The particular case of wave generation in the absence of obstacles by a surface pressure excess has important geophysical and technological applications and has been considered by several authors (Cf.^3,4,8^). For this reason, a section is devoted to this case, where different discontinuous pressure functions are considered, including a constant pressure on a finite support. New results are obtained. We show the existence of privilegied frequencies related to the width of the constant pressure function support for which no outgoing waves are produced. The main steps to obtain the solution are briefly sketched, details may be found in (*I*). In the absence of obstacles, our solution becomes an exact one, and is expressed in closed form. A section including numerical results for the surface pressure of parabolic profile, without or with obstacles, aims to put in evidence the process of energy transmission from the surface pressure excess system to the fluid. Different settings of three circular or elliptical obstacles are considered and the error in satisfying the boundary conditions at the obstacles are tabulated, as these errors are in fact a verification of the accuracy of the proposed method. The level curves of the stream function show that an increase of the surface pressure excess is accompanied by an increase of the velocities.

It is worth noting that the proposed method deals with obstacles having a strict interior, thus it is not applicable to obstacles in the form of plates. Moreover, the numerous numerical experiments have clearly shown that the efficiency of the method decreases for obstacles in the form of slender bodies.

## Problem formulation and frame of reference

**Figure 1:**
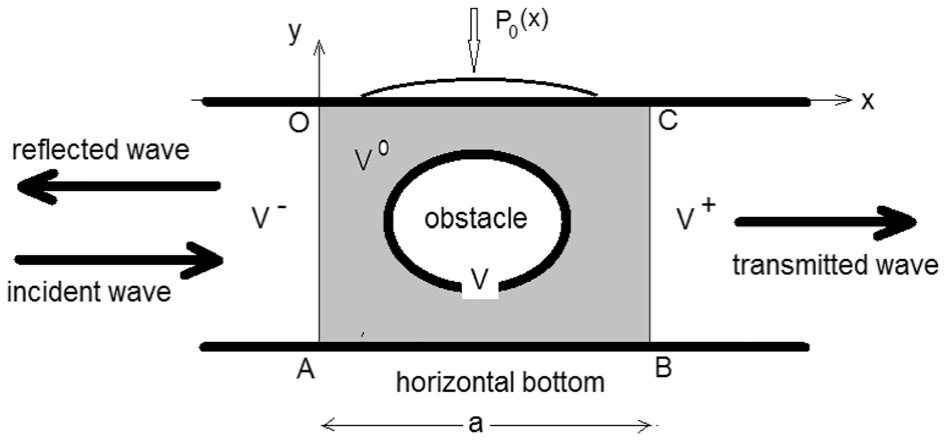
Solution regions for one obstacle in the presence of a surface pressure excess.

Consider the incompressible, two-dimensional free-surface, potential flow of an inviscid fluid of constant density in an infinite channel of constant depth, in the presence of a finite number *P* of submerged obstacles. The flow is bounded from below by an impermeable horizontal bottom. The fluid motion is generated by an incident wave of amplitude $I_0$ and given harmonic time dependence of frequency $\omega$, in addition to a given external pressure excess of the same harmonic time dependence, applied on a finite portion of the free surface. This allows to eliminate time from all the governing equations and boundary conditions, and concentrate only on steady-state solutions to the considered problem. A system of outgoing waves establishes in the fluid body. In the absence of a pressure excess, these are the reflected and the transmitted waves resulting from the incident wave. It is required to determine the resulting fluid motion.

A rectangular frame of reference is used to describe the motion, with origin *O* and *x*-axis along the mean level of the free surface in the direction of propagation of the incident wave, and *y*-axis vertically upwards as in Fig. 1. The time dependent velocity potential $\phi (x,y,t)$ may be written as

1 $$\begin{aligned} \phi (x,y,t)=Re{\left[ \Phi (x,y)e^{-i\omega t}\right] }, \end{aligned}$$

where $\Phi (x,y)$ is a complex function of *x* and *y*.

Within the linearized theory of motion, the system of governing equations and conditions is^1^:

(i) In the fluid mass $\left( -\infty<x<\infty ,\,\,-h\le y\le 0\right)$:
  2 $$\begin{aligned} \frac{\partial ^{2}\Phi }{\partial x^{2}}+\frac{\partial ^{2}\Phi }{\partial y^{2}}=0. \end{aligned}$$
(ii) At the upper bound of the domain of the problem $\left( y=0\right)$: An excess oscillatory external pressure of finite support is applied, having the same order of magnitude and the same harmonic time dependence as those of the incident wave. Even in the absence of the latter ($I_0 = 0$), one can still keep the reflection and the transmission coefficients as describing the waves generated by the excess surface pressure and propagating both upstreams and downstreams. If the external oscillatory pressure excess has the form $P_{ex}(x,t)=P_0(x) e^{-i \omega t}$, the boundary condition at $y=0$ in the frame of the linearized theory is
  3 $$\begin{aligned} \frac{\partial \Phi }{\partial y}-\frac{\omega ^{2}}{g}\Phi = \frac{i \omega }{\rho g} P_0(x). \end{aligned}$$
  Function $P_0(x)$ is assumed to have a finite support $S=\{x: \alpha \le x \le \beta \}$, and is continuous on *S*, except for a finite number of finite discontinuities. Assuming continuity of the surface elevation $\eta (x)$, a jump in the pressure function $P_0(x)$ at a certain point induces a similar behavior of the velocity potential $\Phi$ at the same point.
(iii) On the bottom of the channel $\left( y=-h,\,-\infty<x<\infty \right)$ and on the surfaces of the obtacles:
  4 $$\begin{aligned} \frac{\partial \Phi }{\partial n}=0. \end{aligned}$$
  This condition will be subsequently replaced for convenience by the condition of constancy of the streamfunction $\Psi$ separately at the bottom and on the surfaces of the obstacles.
(iv) The radiation condition at infinity $\left( \left| x\right| \rightarrow \infty \right)$ stating that: no wave is coming from infinity except the prescribed incident one.

The time dependent free surface elevation $\eta ^{*}(x,t)$ and pressure $P^{*}(x,\;y,\;t)$ are:

5 $$\begin{aligned} \eta ^{*}(x,\,t)=Re{\left[ \eta (x)e^{-i\omega t}\right] }, \end{aligned}$$

and

6 $$\begin{aligned} P^{*}(x,y,t)=-\rho gy+Re{\left[ P(x,y)e^{-i\omega t}\right] }, \end{aligned}$$

where $\eta (x)$ and *P*(*x*, *y*) are given in terms of the time independent velocity potential $\Phi (x,y)$ as:

7 $$\begin{aligned} \eta (x)= \frac{i\omega }{ g}\Phi (x,0) - \frac{1 }{\rho g} P_0(x) \end{aligned}$$

and

8 $$\begin{aligned} P\left( x,\,y\right) =i\rho \omega \, \Phi (x,y) + P_0(x). \end{aligned}$$

In what follows we shall set $\rho = 1$ for simplicity.

Certain geophysical phenomena may contain more than one frequency. The mathematical problem in this case will be a superposition a number of subproblems, each of which deals with one frequency only.

## Method of solution

The flow domain is divided into three regions separated by vertical lines as on Fig. 1: A volume $V^0$ for $0 \le x \le a$ containing in its interior the obstacles and the finite support of the pressure excess $(0< \alpha< \beta <a)$, and unbounded volumes $V^-$ to the left and $V^+$ to the right. The solutions in these two semi-infinite regions is obtained by separation of variables and consist of outgoing waves and local perturbations, in addition to an incident wave solution in $V^-$.

We denote by $\lambda _0$ is the positive root of the transcendental equation

9 $$\begin{aligned} \lambda h \,\tanh \lambda h=\frac{\omega ^{2} h}{g} = \kappa , \, \, \text {say}, \end{aligned}$$

and by $\lambda _p, \, p =1,2, \cdots$ the positive roots of the transcendental equation

10 $$\begin{aligned} \lambda h \,\tan \lambda h=-\kappa . \end{aligned}$$

The solution for the velocity potential in the three regions is taken as

11 $$\begin{aligned} \Phi ^{-}(x,y)= & {} \left\{ I_{0}\,e^{i\lambda _{0}x}+R_{0}\,e^{-i\lambda _{0}x}\right\} \cosh \lambda _{0}(y+h) + \sum _{p=1}^{\infty }R_{p}\cos \lambda _{p}(y+h)\,e^{\lambda _{p}x}, \end{aligned}$$

12 $$\begin{aligned} \Phi ^{+}(x,y)= & {} T_{0}\cosh \lambda _{0}(y+h)\,e^{i\lambda _{0}(x-a)}+\sum _{p=1}^{\infty }T_{p}\cos \lambda _{p}(y+h)\,e^{-\lambda _{p}(x-a)}, \end{aligned}$$

13 $$\begin{aligned} \Phi ^{0}(x,y)= & {} \Phi (x,y) + a_0 \left[ \left( y+h \right) ^2 - x^2 \right] + \sum _{k=1}^{2K} a_k \, \ln \frac{r_k(x,y)}{h}. \end{aligned}$$

where $r_k(x,y)$ denotes the norm of vector starting at location $(x_k,y_k)$ outside the flow region and ending at a general point (*x*, *y*) in the fluid. The unknown source strengths $a_k$ will be determined at a later stage so as to satisfy the impermeability condition on the surfaces of the obstacles. The function represented by the summation in the last equation gives the effect of the system of immersed obstacles. It is taken to have vanishing normal derivative at the bottom line, by distributing sources of equal strength symmetrically with respect to the bottom line in the following way:

14 $$\begin{aligned} a_{K+k} = a_k, \quad x_{K+k} = x_k, \quad y_{K+k} = -2h - y_k, \quad k=1,2, \cdots , K. \end{aligned}$$

The coefficient $a_0$ will be defined later on, while the constants $a_k, \quad k=1,2,\cdots ,K$, as stated above, are determined by boundary collocation in satisfying the impermeability conditions on the obstacles surfaces in the form of constancy of the streamfunction on each obstacle separately.

The locations of the above mentioned distribution of sources are taken strictly inside the regions occupied by the obstacles, not belonging to the flow domain. Thus the method is not appropriate for obstacles in the form of plates. Moreover, the efficiency of the method is expected not to be satisfactory in the case of slender obstacles. Cases of flow with immersed vertical plates have been treated by several authors (C.f. Abou-Dina and Helal^10–12^ and Arfaj *et al.*^21^).

The quantities $R_p$ and $T_p$ represent the so-called local perturbations coefficients, necessary to achieve continuity of the flow in the vicinity of the separation vertical lines at $x= 0, a$.

We show here below that the present method of solution allows to incorporate a pressure excess with the same harmonic dependence on time as the incident wave into the procedure and, moreover, produces the exact solution in closed form in the absence of obstacles (C.f. Wehausen and Laitone^4^.

Further considerations will be restricted to the case when a pressure excess of intensity $\rho g h P_0(x)$ is applied to a finite portion of the free surface.

Introduce the finite cosine Fourier transform of the function $P_0(x)$

15 $$\begin{aligned} P_{m}=\int _{0}^{a}\,P_0(x)\,\cos \frac{m \pi x }{a} \,dx,\,m=0,1,2, \cdots \end{aligned}$$

The inversion formula reads

16 $$\begin{aligned} P_0(x)=\sum _{m=0}^{\infty }\frac{2-\delta _{m}^{0}}{a} \,P_{m} \cos \frac{m \pi x}{a}, \end{aligned}$$

where $\delta _{m}^{0}$ is a Krönecker delta symbol. Under the above assumptions, coefficients $P_m$ tend to zero at least like $\displaystyle \frac{1}{m}$ as *m* grows indefinitely large.

The length parameter *a* defined in (*I*) and characterizing the volume $V^0$ must be chosen large enough so as to encompass both the obstacles and the support of the surface pressure excess. It will be given the value

$$\begin{aligned} a = \frac{2 \pi N}{\lambda _0}, \end{aligned}$$

where *N* is a positive integer with undetermined value for the time being.

Apply Green’s second identity to the harmonic functions $\Phi$ and $\bar{\Phi }$, where ‘bar’ denotes the complex conjugate, in a region of the flow extending far away in both horizontal directions. Using the expressions for the velocity potentials far upstreams and downstreams, one finally obtains:

17 $$\begin{aligned} \left|R_0 \right|^2 + \left|T_0 \right|^2 = \left|I_0 \right|^2 + \mathcal {P}, \end{aligned}$$

where

18 $$\begin{aligned} \mathcal {P}= & {} -\frac{4 \omega }{g \left( \sinh 2 \lambda _0 h +2\lambda _0 h \right) }\, \hbox {Re} \int _S \bar{P}_{0} \Phi \bigg |_{y=0} \, dx \nonumber \\= & {} - \frac{4 }{\left( \sinh 2 \lambda _0 h +2\lambda _0 h \right) }\, \hbox {Im} \int _S \bar{P_0} \eta \, dx. \end{aligned}$$

The quantity $\mathcal {P}$ depends, not only on the external pressure function $P_0$, but also on the incident wave intensity through the solution $\Phi$ (or $\eta$). The nonlinear relation (17) illustrates the phenomenon of transmission of energy from the incident wave and the applied oscillatory surface pressure excess to the fluid. Those situations for which

$$\begin{aligned} \mid I_0 \mid ^2 + \mathcal {P} \le 0 \end{aligned}$$

will have no outgoing waves, with the possibility of generation of standing wave in the region $0 \le x \le a$. This aspect will be discussed in more detail in the following section. To the authors knowledge, relation (17) is not mentioned in the literature. For the determination of the function $\Phi (x,y)$, introduce the finite cosine Fourier transform of this function defined as:

19 $$\begin{aligned} \widetilde{\Phi }_{m}(y)=\int _{0}^{a}\,\Phi (x,y)\,\cos \frac{m \pi x }{a} \,dx,\,m=0,1,2, \cdots \end{aligned}$$

The inversion formula reads:

20 $$\begin{aligned} \Phi (x,y)=\sum _{m=0}^{\infty }\frac{2-\delta _{m}^{0}}{a}\widetilde{ \,\Phi _{m}}(y)\cos \frac{m \pi x}{a}, \end{aligned}$$

where $\delta _{m}^{0}$ is a Krönecker delta symbol. Performing this transformation on Laplace’s equation and using conditions of continuity of the velocity potential and its horizontal derivatives at the vertical sections at $x=0,a$ one is finally left with the following expressions for the velocity potential $\Phi ^0$ and the streamfunction $\Psi ^0$:

21 $$\begin{aligned} \Phi ^0(x,y)= & {} \frac{h}{a} \sum _{\begin{array}{c} m=0 \end{array}}^{\infty } \left( 2-\delta _{m}^{0} \right) A_m \frac{\cosh \frac{m \pi }{a}(y+h)}{\cosh \frac{m \pi h}{a}} \cos \frac{m \pi x}{a} \nonumber \\&+ {} \left[ i\left( I_0 - R_0 \right) + \sum _{k=1}^{2K} a_k \alpha _{0k} \right] \cosh \lambda _0 (y+h) \sin \lambda _0 x \nonumber \\&+ {} Z \cosh \lambda _0 (y+h) \cos \lambda _0 x \nonumber \\&+ {} \sum _{p=1}^{\infty } \left( R_p + \sum _{k=1}^{2K} a_k \alpha _{pk} \right) \frac{\cosh \lambda _p (a-x)}{\sinh \lambda _p a} \cos \lambda _p (y+h) \nonumber \\& + {} \sum _{p=1}^{\infty } \left( T_p + \sum _{k=1}^{2K} a_k \beta _{pk} \right) \frac{\cosh \lambda _p x}{\sinh \lambda _p a} \cos \lambda _p (y+h) \nonumber \\&+ {} a_0 \left[ \left( y+h \right) ^2 - x^2 \right] + \sum _{k=1}^{2K} a_k \, \ln \frac{r_k(x,y)}{d}, \end{aligned}$$

22 $$\begin{aligned} \Psi ^0(x,y)= & {} - \frac{h}{a} \sum _{\begin{array}{c} m=0 \end{array}}^{\infty } \left( 2-\delta _{m}^{0} \right) A_m \frac{\sinh \frac{m \pi }{a}(y+h)}{\cosh \frac{m \pi h}{a}} \sin \frac{m \pi x}{a} \nonumber \\&+ {} \left[ i\left( I_0 - R_0 \right) + \sum _{k=1}^{2K} a_k \alpha _{0k} \right] \sinh \lambda _0 (y+h) \cos \lambda _0 x \nonumber \\&- {} Z \sinh \lambda _0 (y+h) \sin \lambda _0 x \nonumber \\&- {} \sum _{p=1}^{\infty } \left( R_p + \sum _{k=1}^{2K} a_k \alpha _{pk} \right) \frac{\sinh \lambda _p (a-x)}{\sinh \lambda _p a} \sin \lambda _p (y+h) \nonumber \\&+ {} \sum _{p=1}^{\infty } \left( T_p + \sum _{k=1}^{2K} a_k \beta _{pk} \right) \frac{\sinh \lambda _p x }{\sinh \lambda _p a} \sin \lambda _p (y+h) \nonumber \\&- {} 2a_0 x \left( y+h \right) + \sum _{k=1}^{2K} a_k \arctan \frac{y - y_k}{x - x_k}. \end{aligned}$$

where

23 $$\begin{aligned} A_m \left( \frac{m \pi h}{a} \tanh \frac{m \pi h}{a} - \kappa \right) = a_0 H_m + i \frac{a}{h} \left( \omega h^2 \right) \frac{P_m}{a} + \sum _{k=1}^{2K} a_k J_{mk}, \end{aligned}$$

with

24 $$\begin{aligned} a_0 = \frac{1}{h^2} \left[ - i \frac{h}{a} \left( \omega h^2 \right) \frac{a P_{2N}}{H_{2N}} - \left( \frac{h}{a} \right) ^2 \sum _{k=1}^{2K} a_k \frac{a^2 J_{2N,k}}{H_{2N}} \right] . \end{aligned}$$

In case of no obstacles, the last condition provides the exact value of $a_0$:

25 $$\begin{aligned} a_0 = - i \frac{h}{a} \frac{1}{h^2} \left( \omega h^2 \right) \frac{a P_{2N}}{H_{2N}}. \end{aligned}$$

It is important to note that the expression (22) for the streamfunction satisfies the condition of constancy of this function at the bottom topography.

For convenience, we shall write the coefficient $A_m$ as a sum of two terms, representing the contributions to the solution of the surface pressure excess and the obstacles respectively:

26 $$\begin{aligned} A_m = B_m + \sum _{k=1}^{2K} a_k B_{mk}, \end{aligned}$$

where

27 $$\begin{aligned} B_m= & {} i \frac{a}{h} \left( \omega ^2h \right) \frac{1}{\frac{m \pi h}{a} \tanh \frac{m \pi h}{a} - \kappa } \left( - \frac{1}{a} \frac{ P_{2N}}{H_{2N}} H_m + \frac{P_m}{a} \right) \nonumber \\ B_{mk}= & {} \frac{1}{\frac{m \pi h}{a} \tanh \frac{m \pi h}{a} - \kappa } \left( J_{mk} - \frac{J_{2N,k}}{H_{2N}} H_m \right) . \end{aligned}$$

Also,

28 $$\begin{aligned} R_0= & {} \frac{1}{2J_0} \frac{h}{a} \sum _{\begin{array}{c} m=0 \end{array}}^{\infty } \left( 2-\delta _{m}^{0} \right) B_m \frac{1-(-1)^m}{\cosh \frac{m \pi h}{a}} I_{0m} - \frac{1}{2J_0} \frac{a}{h} \frac{a P_{2N}}{H_{2N}} i \left( \omega h^2 \right) \frac{1}{\lambda _0 h} \sinh \lambda _0 h \nonumber \\&+ {} \sum _{k=1}^{2K} a_k \left[ \frac{1}{2J_0} \frac{h}{a} \sum _{\begin{array}{c} m=0 \\ m \ne 2N \end{array}}^{\infty } \left( 2-\delta _{m}^{0} \right) B_{mk} \frac{1-(-1)^m}{\cosh \frac{m \pi h}{a}} I_{0m} + \frac{1}{2J_0} \left( S_k - V_k \right) - \frac{i}{2} \left( \alpha _{0k} + \beta _{0k} \right) \right. \nonumber \\&- {} \left. \frac{1}{2J_0} \frac{a^2 J_{2N,k}}{H_{2N}} \frac{1}{\lambda _0 h} \sinh \lambda _0 h \right] \end{aligned}$$

29 $$\begin{aligned} T_0= & {} I_0 - \frac{1}{2J_0} \frac{h}{a} \sum _{\begin{array}{c} m=0 \end{array}}^{\infty } \left( 2-\delta _{m}^{0} \right) B_m \frac{1-(-1)^m}{\cosh \frac{m \pi h}{a}} I_{0m} + \frac{1}{2J_0} \frac{a}{h} \frac{a P_{2N}}{H_{2N}} i \left( \omega h^2 \right) \frac{1}{\lambda _0 h} \sinh \lambda _0 h \nonumber \\&- {} \sum _{k=1}^{2K} a_k \left[ \frac{1}{2J_0} \frac{h}{a} \sum _{\begin{array}{c} m=0 \\ m \ne 2N \end{array}}^{\infty } \left( 2-\delta _{m}^{0} \right) B_{mk} \frac{1-(-1)^m}{\cosh \frac{m \pi h}{a}} I_{0m} + \frac{1}{2J_0} \left( S_k - V_k \right) + \frac{i}{2} \left( \alpha _{0k} + \beta _{0k} \right) \right. \nonumber \\&- {} \left. \frac{1}{2J_0} \frac{a^2 J_{2N,k}}{H_{2N}} \frac{1}{\lambda _0 h} \sinh \lambda _0 h \right] , \end{aligned}$$

30 $$\begin{aligned} Z= & {} I_0 - \frac{1}{2J_0} \frac{h}{a} \sum _{\begin{array}{c} m=0 \end{array}}^{\infty } \left( 2-\delta _{m}^{0} \right) A_m \frac{1 + (-1)^m}{\cosh \frac{m \pi h}{a}} I_{0m} + \frac{1}{2J_0} a_0 \left( 2S_0 - \frac{a^2 }{\lambda _0 h} \sinh \lambda _0 h \right) \nonumber \\&- {} \frac{1}{2J_0} \sum _{k=1}^{2K} a_k \left( S_k + V_k \right) - \frac{i}{2} \sum _{k=1}^{2K} a_k \left( \alpha _{0k} + \beta _{0k} \right) \end{aligned}$$

and two linear algebraic equations for the determination of the coefficients $R_p, T_p$:

31 $$\begin{aligned}{} & {} \frac{1}{J_p} \frac{h}{a} \sum _{\begin{array}{c} m=0 \end{array}}^{\infty } \left( 2-\delta _{m}^{0} \right) A_m \frac{1}{\cosh \frac{m \pi h}{a}} I_{mp} + \sum _{k=1}^{2K} a_k \alpha _{pk} \frac{\cosh \lambda _p a}{\sinh \lambda _p a} \nonumber \\&+ {} \sum _{k=1}^{2K} a_k \beta _{pk} \frac{1}{\sinh \lambda _p a} + \frac{1}{J_p} a_0 U_p + \frac{1}{J_p} \sum _{k=1}^{2K} a_k S_{kp} \nonumber \\&= {} \left( 1 - \frac{\cosh \lambda _p a}{\sinh \lambda _p a} \right) R_{p} - \frac{1}{\sinh \lambda _p a} T_p. \end{aligned}$$

32 $$\begin{aligned}{} & {} \frac{1}{J_p} \frac{h}{a} \sum _{\begin{array}{c} m=0 \end{array}}^{\infty } \left( 2-\delta _{m}^{0} \right) (-1)^m A_m \frac{1}{\cosh \frac{m \pi h}{a}} I_{mp} + \sum _{k=1}^{2K} a_k \alpha _{pk} \frac{1}{\sinh \lambda _p a} \nonumber \\&+ {} \sum _{k=1}^{2K} a_k \beta _{pk} \frac{\cosh \lambda _p a}{\sinh \lambda _p a} + \frac{1}{J_p} a_0 \left( U_p - \frac{a^2}{\lambda _p} \sin \lambda _p h \right) + \frac{1}{J_p} \sum _{k=1}^{2K} a_k V_{kp} \nonumber \\&= {} - \frac{1}{\sinh \lambda _p a} R_p + \left( 1 - \frac{\cosh \lambda _p a}{\sinh \lambda _p a} \right) T_{p} \end{aligned}$$

The determinant $\Delta _p$ of this system of two linear algebraic equations for $R_p$ and $T_p$ is given by:

33 $$\begin{aligned} \Delta _p = \left( 1 - \frac{\cosh \lambda _p a}{\sinh \lambda _p a} \right) ^2 - \frac{1}{\sinh ^2 \lambda _p a} = 2 \left( 1 - \coth \lambda _p a \right) = -4 \frac{e^{-2 \lambda _p a}}{1- e^{-2 \lambda _p a}} \end{aligned}$$

and this tends to zero exponentially as $p \rightarrow \infty$. This implies that both coefficients $R_p$ and $T_p$ will have a factor of $e^{\lambda _p a}$ in their expressions. Referring to expressions (11) and (12) for the solution, one finds out that the effect of the local perturbations will start to strictly decrease only when $x < -a$ and $x > 2a$.

It is important to note here that the expression (30) for *Z* is not obtained directly, but rather through a limiting process as $\lambda _0 a \rightarrow 2 \pi N$. We have omitted the details for conciseness.

One verifies that

34 $$\begin{aligned} R_0 + T_0 = I_0 - i \sum _{k=1}^{2K} a_k \left( \alpha _{0k} + \beta _{0k} \right) . \end{aligned}$$

The terms involving the coefficients $a_k$ in the expressions (28) and (29) for the reflection and the transmission coefficients vanish in the absence of obstacles. The coefficient $A_{2N}$ cannot be determined directly from (23) as the multiplying coefficient vanishes. It needs to be calculated by a limiting procedure as $m \rightarrow 2N$. The obtained value is listed below. All coefficients appearing in the above equations are given by the expressions:

$$\begin{aligned} \alpha _{0k}= & {} \frac{1}{J_0 \lambda _0 h} \int _{-h}^{0} \frac{x_k \cosh \lambda _0 (y+h)}{(y-y_k)^2 +x_k^2} \, dy, \quad \alpha _{pk} = \frac{1}{J_p \lambda _p h} \int _{-h}^{0} \frac{x_k \cos \lambda _p (y+h)}{(y-y_k)^2 +x_k^2} \, dy,\\ \beta _{0k}= & {} \frac{1}{J_0 \lambda _0 h} \int _{-h}^{0} \frac{(a - x_k) \cosh \lambda _0 (y+h)}{(y-y_k)^2 +(a-x_k)^2} \, dy, \quad \beta _{pk} = \frac{1}{J_p \lambda _p h} \int _{-h}^{0} \frac{(a - x_k) \cos \lambda _p (y+h)}{(y-y_k)^2 +(a-x_k)^2} \, dy,\\ I_{0m}= & {} \int _{-h}^0 \cosh \frac{m \pi }{a}(y+h) \cosh \lambda _0 (y+h) \, dy \\= & {} \frac{h}{1 - \frac{m^2 \pi ^2}{\lambda _0^2 a^2}} \left[ \frac{1}{\lambda _0 h} \cosh \frac{m \pi h}{a} \sinh \lambda _0 h - \frac{1}{(\lambda _0 h)^2} \frac{m \pi h}{a} \sinh \frac{m \pi h}{a} \cosh \lambda _0 h \right] ,\\ I_{mp}= & {} \int _{-h}^0 \cosh \frac{m \pi }{a}(y+h) \cos \lambda _p (y+h) \, dy \\= & {} \frac{h}{1 + \frac{m^2 \pi ^2}{\lambda _p^2 a^2}} \left[ \frac{1}{\lambda _p h} \cosh \frac{m \pi h}{a} \sin \lambda _p h + \frac{1}{(\lambda _p h)^2} \frac{m \pi h}{a} \sinh \frac{m \pi h}{a} \cos \lambda _p h \right] ,\\ J_0= & {} \frac{1}{h} \int _{-h}^{0}\,\cosh ^2 \lambda _0(\sigma +h) \, d\sigma = \frac{1}{2} \left( 1 + \frac{ \sinh 2 \lambda _0 h}{2 \lambda _0 h} \right) , \\ J_p= & {} \frac{1}{h} \int _{-h}^{0}\,\cos ^2 \lambda _p(\sigma +h) \, d\sigma = \frac{1}{2} \left( 1 + \frac{ \sin 2 \lambda _p h}{2 \lambda _p h} \right) ,\\ H_m= & {} \int _0^a \left[ -2h + \frac{\omega ^2}{g} \left( h^2 - \sigma ^2 \right) \right] \cos \frac{m \pi \sigma }{a} \, d\sigma = -2 \kappa \frac{a}{h} (-1)^{m+1} \frac{a^2}{m^2 \pi ^2}, \\ J_{mk}= & {} \int _0^{\frac{a}{h}} \frac{\frac{y_k}{h}}{\left( \sigma - \frac{x_k}{h} \right) ^2+ (\frac{y_k}{h})^2} \, \cos \frac{m \pi h \sigma }{a} \, \, d\sigma + \kappa \int _0^{\frac{a}{h}} \ln \sqrt{\left( \sigma - \frac{x_k}{h} \right) ^2+ (\frac{y_k}{h})^2} \, \cos \frac{m \pi h \sigma }{a} \, \, d\sigma \\ A_{2N}= & {} \frac{a}{\pi h} \frac{1}{\left( \tanh \lambda _0 h + \lambda _0 h \, \text{ sech}^2 \lambda _0 h \right) } \left( a_0 H^{(1)} + i \omega h^2 \frac{1}{h} P^{(1)} + \sum _{k=1}^{2K} a_k J_{k}^{(1)} \right) . \end{aligned}$$

A careful examination of the series in the expression for $\Phi _0$ reveals that they are convergent. Using the second mean-value theorem for integrals, coefficient $J_{mk}$ and $K_{mk}$ listed above are shown to be of the order $\frac{1}{m}$, hence the general term of the first series intervening in (21) for $\Phi _0$ is of the order $\displaystyle \frac{1}{m^2} \, e^{\frac{-m \pi \vert y \vert }{a}}$ as *m* increases indefinitely. The other series are convergent as well.

At this stage, all the boundary conditions of the problem have been satisfied rigorously, except the impermeability condition on the surfaces of the obstacles. For convenience, this condition is replaced with the condition of constancy of the streamfunction on each obstacle separately. These constant values, say $C_p, p=1,2, \cdots P$, on the p-th obstacle, are taken as additional unknowns of the problem, together with the unknown coefficients $a_k, \, k = 1, 2, \cdots K$ embedded in the expression (13) for the solution in region $V^0$. As explained in (*I*), the determination of all the unknown coefficients and constants will be achieved in an approximate way by boundary collocation on the obstacles surfaces. As a result, one obtains a system of linear algebraic equations, the solution of which provides the required values. This completes the determination of the semi-analytical approximate solution.

The errors in satisfying the condition of constancy of the streamfunction separately on the obstacles are defined as:

35 $$\begin{aligned} E_p = \text {max} \left| \Psi - C_p\right| , \quad p=1,2, \cdots P, \end{aligned}$$

the maximum being taken on all points of the surface of the p-th obstacle. These errors are in fact a measure of the efficiency of the proposed method.

The above solution may be analyzed in the shallow water approximation by making the replacement $\lambda _0 h = \sqrt{\kappa }$, or within the deep water approximation by taking $\lambda _0 h = \kappa$.

## Exact solutions in the absence of obstacles

In the absence of obstacles, our solution becomes an exact one and is expressed in closed form. It is obtained by setting $a_k = 0, \, k = 1, 2, \cdots K$. The solutions in the semi-infinite regions are still given by (11) and (12). As to the exact solution in the region $V^0$, it is given by (13) after making the mentioned substitutions.

When there is no external surface pressure excess, then $A_m = a_0 = 0, \, m = 1, 2, \cdots$ and one gets $R_0 = 0$ and $T_0 = I_0$.

When there is no incident wave, the wave amplitudes at negative and positive infinities are given by the formulae:

$$\begin{aligned} R_0= & {} \frac{1}{2J_0} \frac{h}{a} \sum _{\begin{array}{c} m=0 \end{array}}^{\infty } \left( 2-\delta _{m}^{0} \right) B_m \frac{1-(-1)^m}{\cosh \frac{m \pi h}{a}} I_{0m} - \\{} & {} \frac{1}{2J_0} \frac{a}{h} \frac{a P_{2N}}{H_{2N}} i \left( \omega h^2 \right) \frac{1}{\lambda _0 h} \sinh \lambda _0 h - \frac{1}{2J_0} \frac{a^2 J_{2N,k}}{H_{2N}} \frac{1}{\lambda _0 h} \sinh \lambda _0 h = - T_0, \end{aligned}$$

The Fourier coefficients of even rank of the applied pressure function $P_0(x)$, with the exception of $P_{2N}$, do not contribute towards the values of $R_0$ and $T_0$. As to the coefficients of local perturbations, they are given in terms of the totality of the coefficients $P_m, \, m=0,1,2, \cdots$ as:

36 $$\begin{aligned} R_p= & {} \frac{1}{J_p} \frac{h}{a} \sum _{\begin{array}{c} m=0 \end{array}}^{\infty } \left( 2-\delta _{m}^{0} \right) (-1)^m A_m \frac{\sinh \lambda _p a}{\cosh \frac{m \pi h}{a}} I_{mp} \frac{(-1)^m + \sinh \lambda _p a - \cosh \lambda _p a}{\cosh 2 \lambda _p a - \sinh 2 \lambda _p a - 1} \nonumber \\&+ {} \frac{1}{J_p} a_0 \left( U_p - \frac{a^2}{\lambda _p} \sin \lambda _p h \right) \frac{\sinh \lambda _p a}{\cosh 2 \lambda _p a - \sinh 2 \lambda _p a - 1} \nonumber \\&+ {} \frac{1}{J_p} a_0 U_p \sinh \lambda _p a \frac{\sinh \lambda _p a - \cosh \lambda _p a}{\cosh 2 \lambda _p a - \sinh 2 \lambda _p a - 1},\end{aligned}$$

37 $$\begin{aligned} T_p= & {} \frac{1}{J_p} \frac{h}{a} \sum _{\begin{array}{c} m=0 \end{array}}^{\infty } \left( 2-\delta _{m}^{0} \right) A_m \frac{\sinh \lambda _p a}{\cosh \frac{m \pi h}{a}} I_{mp} \, \frac{(-1)^m \left( \sinh \lambda _p a - \cosh \lambda _p a \right) +1}{\cosh 2 \lambda _p a - \sinh 2 \lambda _p a - 1} \nonumber \\&+ {} \frac{1}{J_p} a_0 \left( U_p - \frac{a^2}{\lambda _p} \sin \lambda _p h \right) \frac{\sinh \lambda _p a \left( \sinh \lambda _p a - \cosh \lambda _p a \right) }{\cosh 2 \lambda _p a - \sinh 2 \lambda _p a - 1} \nonumber \\&+ {} \frac{1}{J_p} a_0 U_p \frac{1}{\cosh 2 \lambda _p a - \sinh 2 \lambda _p a - 1}, \end{aligned}$$

while the expression of coefficient *Z* introduced above simplifies to:

$$\begin{aligned} Z= & {} - \frac{1}{2J_0} \frac{h}{a} \sum _{\begin{array}{c} m=0 \end{array}}^{\infty } \left( 2-\delta _{m}^{0} \right) A_m \frac{1 + (-1)^m}{\cosh \frac{m \pi h}{a}} I_{0m} + \frac{1}{2J_0} a_0 \left( 2S_0 - \frac{a^2 }{\lambda _0 h} \sinh \lambda _0 h \right) . \end{aligned}$$

These results clearly show the effect of a harmonic surface pressure excess applied to a finite portion of the free surface in generating waves in both directions, which, in turn, means absorption of energy by the fluid. The velocity potential and the streamfunction in the volume $V^0$ are, respectively:

38 $$\begin{aligned} \Phi ^0(x,y)= & {} \frac{h}{a} \sum _{\begin{array}{c} m=0 \end{array}}^{\infty } \left( 2-\delta _{m}^{0} \right) A_m \frac{\cosh \frac{m \pi }{a}(y+h)}{\cosh \frac{m \pi h}{a}} \cos \frac{m \pi x}{a} \nonumber \\&- {} i R_0 \cosh \lambda _0 (y+h) \sin \lambda _0 x + Z \cosh \lambda _0 (y+h) \cos \lambda _0 x \nonumber \\&+ {} \sum _{p=1}^{\infty } R_p \frac{\cosh \lambda _p (a-x)}{\sinh \lambda _p a} \cos \lambda _p (y+h) \nonumber \\&+ {} \sum _{p=1}^{\infty } T_p \frac{\cosh \lambda _p x}{\sinh \lambda _p a} \cos \lambda _p (y+h) + a_0 \left[ \left( y+h \right) ^2 - x^2 \right] \end{aligned}$$

and

39 $$\begin{aligned} \Psi ^0(x,y)= & {} - \frac{h}{a} \sum _{\begin{array}{c} m=0 \end{array}}^{\infty } \left( 2-\delta _{m}^{0} \right) A_m \frac{\sinh \frac{m \pi }{a}(y+h)}{\cosh \frac{m \pi h}{a}} \sin \frac{m \pi x}{a} \nonumber \\&- {} i R_0 \sinh \lambda _0 (y+h) \cos \lambda _0 x - Z \sinh \lambda _0 (y+h) \sin \lambda _0 x \nonumber \\- & {} \sum _{p=1}^{\infty } R_p \frac{\sinh \lambda _p (a-x)}{\sinh \lambda _p a} \sin \lambda _p (y+h) \nonumber \\&+ {} \sum _{p=1}^{\infty } T_p \frac{\sinh \lambda _p x }{\sinh \lambda _p a} \sin \lambda _p (y+h) - 2a_0x \left( y + h \right) \end{aligned}$$

The first three terms in the velocity potential function are responsible for producing a system of progressive waves travelling in both directions in $V^0$. In those cases when there are no outgoing waves in the semi-infinite regions, a standing wave establishes in $V^0$. This will be investigated in more detail in the coming special cases of the surface pressure function. The last three terms in the expression for the velocity potential represent local perturbations.

### The case of a constant pressure excess

Subject to the above limitations, the present formulation remains valid for any pressure distribution as long as it satisfies $P_0(0)= P_0(a) = 0$ ensuring continuity of the physical quantities, in particular the pressure and velocity field, as well the free surface elevation. This includes the case of a constant pressure with finite support lying in $0< x < a$. When $P_0(x) = A (= const.)$ on the interval $0< \alpha \le x \le \beta < a$ and vanishes otherwise, one has

$$\begin{aligned} P_0 = \left( \beta - \alpha \right) A, \qquad P_m = A \, \frac{\sin \frac{m \pi \beta }{a} - \sin \frac{m \pi \alpha }{a}}{ m \pi }, \quad m \ge 1. \end{aligned}$$

The case $\alpha = 0$ and $\beta = a$ needs special attention, and may be treated as a limiting case from the above. It yields

$$\begin{aligned} P_0 = A, \qquad P_m = 0, \quad m \ge 1, \end{aligned}$$

so that

40 $$\begin{aligned} R_0 = T_0 = 0. \end{aligned}$$

This is the solution without outgoing waves noticed earlier by Stoker^3^ and Sarmento and Falc$\tilde{a}$o [?]. The velocity potential and the streamfunction for this case are:

41 $$\begin{aligned} \Phi ^0(x,y)= & {} - \frac{2}{J_0} \frac{h}{a} \left( - \frac{1}{\kappa } i \omega h a I_{00} P_0 + \frac{1}{\cosh \frac{2N \pi h}{a}} B_{2N} I_{0,2N} \right) \cosh \lambda _0 (y+h) \cos \lambda _0 x \nonumber \\{} & {} + \sum _{p=1}^{\infty } R_p \frac{1}{\sinh \lambda _p a} \left[ \cosh \lambda _p x + \cosh \lambda _p (a-x) \right] \cos \lambda _p (y+h) \end{aligned}$$

and

42 $$\begin{aligned} \Psi ^0(x,y)= & {} \frac{2}{J_0} \frac{h}{a} \left( - \frac{1}{\kappa } i \omega h a I_{00} P_0 + \frac{1}{\cosh \frac{2N \pi h}{a}} B_{2N} I_{0,2N} \right) \sinh \lambda _0 (y+h) \sin \lambda _0 x\nonumber \\&+ {} \sum _{p=1}^{\infty } R_p \frac{1}{\sinh \lambda _p a} \left[ \sinh \lambda _p x - \sinh \lambda _p (a-x) \right] \sin \lambda _p (y+h), \end{aligned}$$

with

$$\begin{aligned} R_p = - \frac{1}{J_p} \frac{h}{a} B_0 \, I_{0p} \frac{\sinh \lambda _p a}{\cosh \lambda _p a - \sinh \lambda _p a + 1 } = T_p. \end{aligned}$$

The expression (42) for $\Psi ^0$ is composed of two distinct parts: The second part involving the infinite series represents local perturbations, while the first part vanishing at $x=0$ , $x=a$ and the bottom line represents a standing wave. The amplitude of this standing wave is easily seen to be

$$\begin{aligned} \text {Amp} = \frac{2}{J_0} \frac{h}{a} \left|- \frac{1}{\kappa } i \omega h a I_{00} P_0 + \frac{1}{\cosh \frac{2N \pi h}{a}} B_{2N} I_{0,2N} \right|\cosh \lambda _0 h \end{aligned}$$

### Miscellaneous cases for the surface pressure excess

1. When the pressure function is of the form
  43 $$\begin{aligned} P_0(x) = P_{2N} \cos \frac{2N \pi x}{a}, \end{aligned}$$
  one has
  44 $$\begin{aligned} R_0 = - \frac{1}{2J_0} i \omega h a \frac{P_{2N}}{H_{2N}} \frac{a^2 h}{\lambda _0 h} \sinh \lambda _0 h = - T_0 \end{aligned}$$
  and
  45 $$\begin{aligned} R_p= & {} \frac{1}{J_p} i \omega h a \frac{P_{2N}}{H_{2N}} \left( U_p - \frac{a^2 h}{\lambda _p h} \sin \lambda _p h \right) \frac{\sinh \lambda _p a}{\cosh 2 \lambda _p a - \sinh 2 \lambda _p a - 1} \nonumber \\&+ {} \frac{1}{J_p} i \omega h a \frac{P_{2N}}{H_{2N}} U_p \sinh \lambda _p a \frac{\sinh \lambda _p a - \cosh \lambda _p a}{\cosh 2 \lambda _p a - \sinh 2 \lambda _p a - 1}, \end{aligned}$$
  46 $$\begin{aligned} T_p= & {} \frac{1}{J_p} i \omega h a \frac{P_{2N}}{H_{2N}} \left( U_p - \frac{a^2 h}{\lambda _p h} \sin \lambda _p h \right) \frac{\sinh \lambda _p a \left( \sinh \lambda _p a - \cosh \lambda _p a \right) }{\cosh 2 \lambda _p a - \sinh 2 \lambda _p a - 1} \nonumber \\&+ {} \frac{1}{J_p} i \omega h a \frac{P_{2N}}{H_{2N}} U_p \frac{1}{\cosh 2 \lambda _p a - \sinh 2 \lambda _p a - 1}, \end{aligned}$$
  and
  47 $$\begin{aligned} Z = \frac{1}{2J_0} i \omega h a \frac{P_{2N}}{H_{2N}} \left( 2S_0 - \frac{a^2}{\lambda _0} \sinh \lambda _0 h \right) , \end{aligned}$$
  with
  $$\begin{aligned} S_0= & {} \frac{1}{h} \int _{-h}^0 \left( y+h \right) ^2 \cosh \lambda _0 (y+h) \, dy = h^2 \left[ \frac{2 + \lambda _0^2 h^2}{(\lambda _0 h)^3} \sinh \lambda _0 h - \frac{2}{(\lambda _0 h)^2} \cosh \lambda _0 h \right] \\ S_k= & {} \int _{-1}^0 \ln r_k \left( 0,\frac{\sigma }{h} \right) \cosh \left[ \lambda _0 h ( \sigma +1) \right] \, d \sigma \\ V_k= & {} \int _{-1}^0 \ln r_k \left( \frac{a}{h},\frac{\sigma }{h} \right) \cosh \left[ \lambda _0 h ( \sigma +1) \right] \, d \sigma \\ U_p= & {} \int _{-h}^0 \left( y + h \right) ^2 \cos \lambda _p (y+h) \, dy = h^3 \left[ - \frac{2 - \lambda _p^2 h^2}{(\lambda _p h)^3} \sin \lambda _p h + \frac{2}{(\lambda _p h)^2} \cos \lambda _p h \right] \\ S_{kp}= & {} h \int _{-1}^0 \ln r_k \left( 0,\frac{\sigma }{h} \right) \cos \left[ \lambda _p h ( \sigma +1) \right] \, d \sigma \\ V_{kp}= & {} h \int _{-1}^0 \ln r_k \left( \frac{a}{h},\frac{\sigma }{h} \right) \cos \left[ \lambda _p h ( \sigma +1) \right] \, d \sigma \\ H^{(1)}= & {} \lambda _0 \int _0^{a} \sigma ^3 \sin \lambda _0 \sigma \, d\sigma = \frac{4 \pi N h^3}{(\lambda _0 h)^3} \left( 3 - 2 \pi ^2 N^2 \right) , \\ P^{(1)}= & {} - \lambda _0 h \int _{0}^{\frac{a}{h}}\, h \sigma P_0(h \sigma )\,\sin \lambda _0 h \sigma \,d\sigma , \\ J_{k}^{(1)}= & {} - \int _0^{\frac{a}{h}} \frac{y_k}{\left( \sigma - x_k \right) ^2+ y_k^2} \, \sin \lambda _0 \sigma \, \, d\sigma \\- & {} \kappa \int _0^{\frac{a}{h}} \ln \sqrt{\left( \sigma - x_k \right) ^2+ y_k^2} \, \sin \lambda _0 h \sigma \, \, d\sigma . \end{aligned}$$
  The velocity potential and the streamfunction are given by (41) and (42) with
  48 $$\begin{aligned} B_m \left( \frac{m \pi h}{a} \tanh \frac{m \pi h}{a} - \kappa \right) = - i \omega h a \frac{P_{2N}}{H_{2N}} H_m. \end{aligned}$$
  The flow in this case is composed of three components: Outgoing waves in the semi-infinite regions with amplitude $|R_0 \cosh \lambda _0 h |$, a system of progressive waves travelling in both directions in the region bounded by the vertical sections at $x=0$ and $x=a$, in addition to local perturbations dying out far from the region $V^0$.
2. The pressure function is of the form
  49 $$\begin{aligned} P_0(x) = g h P_{2Nr} \cos \frac{2Nr \pi x}{a}, \quad r=2,3, \cdots \end{aligned}$$
  50 $$\begin{aligned} R_0 = T_0 = 0, \end{aligned}$$
  with
  51 $$\begin{aligned}{} & {} A_{2Nr} \left( \frac{2Nr \pi h}{a} \tanh \frac{2Nr \pi h}{a} - \kappa \right) = i \omega h a P_{2Nr}, \, A_m =0, m \ne 2Nr, \end{aligned}$$
  52 $$\begin{aligned} Z= & {} - \frac{2}{J_0} \frac{h}{a} \frac{1}{\cosh \frac{2Nr \pi h}{a}} A_{2Nr} I_{0,2Nr}. \end{aligned}$$
  53 $$\begin{aligned} \Phi ^0(x,y)= & {} \frac{2h}{a} A_{2Nr} \frac{\cosh \frac{2Nr \pi }{a}(y+h)}{\cosh \frac{2Nr \pi h}{a}} \cos \frac{2Nr \pi x}{a} \nonumber \\+ & {} Z \cosh \lambda _0 (y+h) \cos \lambda _0 x \nonumber \\&+ {} \sum _{p=1}^{\infty } R_p \frac{\cosh \lambda _p (a-x)}{\sinh \lambda _p a} \cos \lambda _p (y+h) \nonumber \\&+ {} \sum _{p=1}^{\infty } T_p \frac{\cosh \lambda _p x}{\sinh \lambda _p a} \cos \lambda _p (y+h) + a_0 \left[ \left( y+h \right) ^2 - x^2 \right] \end{aligned}$$
  and
  54 $$\begin{aligned} \Psi ^0(x,y)= & {} - \frac{2h}{a}A_{2Nr} \frac{\sinh \frac{2Nr \pi }{a}(y+h)}{\cosh \frac{2Nr \pi h}{a}} \sin \frac{2Nr \pi x}{a} \nonumber \\&- {} Z \sinh \lambda _0 (y+h) \sin \lambda _0 x \nonumber \\&- {} \sum _{p=1}^{\infty } R_p \frac{\sinh \lambda _p (a-x)}{\sinh \lambda _p a} \sin \lambda _p (y+h) \nonumber \\&+ {} \sum _{p=1}^{\infty } T_p \frac{\sinh \lambda _p x }{\sinh \lambda _p a} \sin \lambda _p (y+h) - 2a_0x \left( y + h \right) . \end{aligned}$$
3. The case when the pressure function is a delta function concentrated at $x_0 (< a)$, i.e. the case of a point pressure applied at $x_0$, was noted by Stoker^3^. Here:
  $$\begin{aligned} P_m = \cos \frac{m \pi x_0}{a}, \quad \forall m \ge 0, \end{aligned}$$
  the flow characteristics may be obtained from the general formulae presented above after performing the proper substitutions. Unlike other cases mentioned above, here there will always be outgoing waves.

## Numerical applications

**Figure 2:**
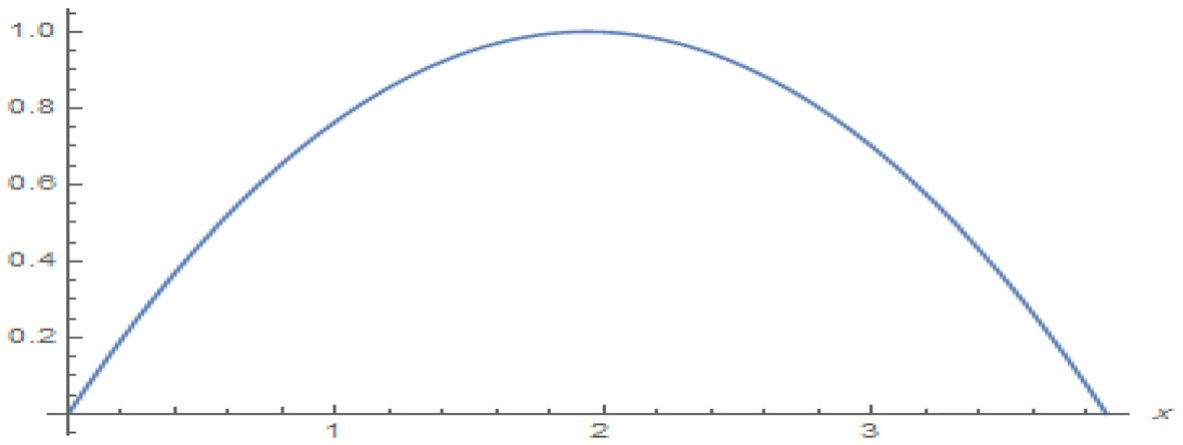
Parabolic profile of the surface pressure excess with support [0, *a*] for $P_{max}=1.0$ and $a=3.87$.

**Figure 3:**
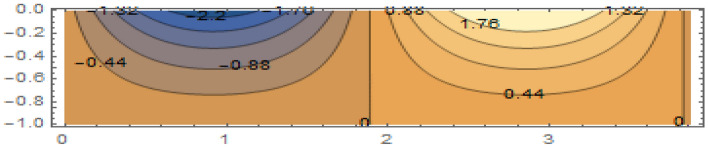
Streamlines for $P_{max}=1$ and $a=3.87$ in region $V^0$.

**Figure 4:**
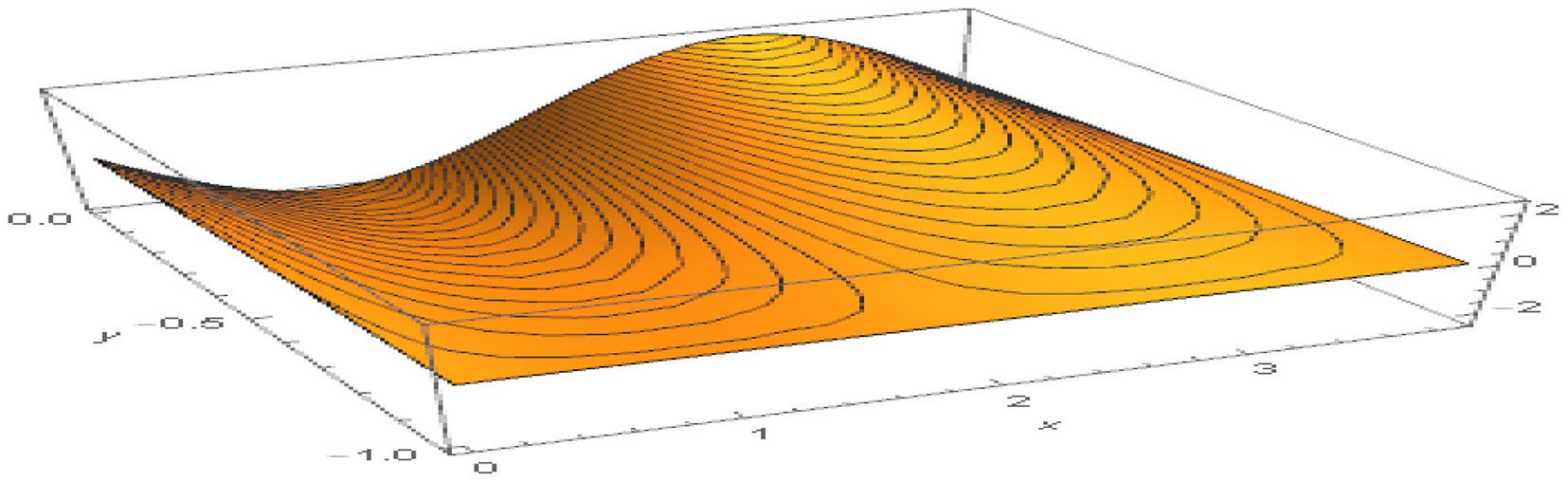
Streamfunction and level curves for $P_{max}=1$ and $a=3.87$ in region $V^0$.

**Figure 5:**
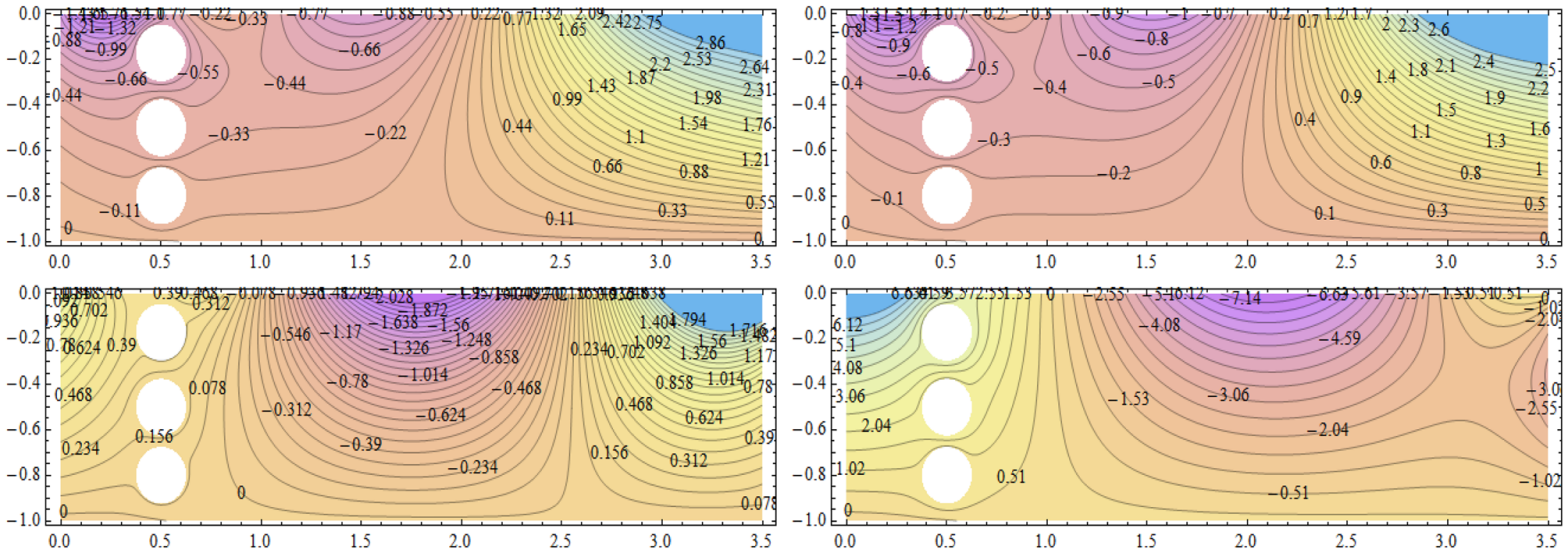
Streamfunction and level curves for three circular obstacles in vertical setting. Obstacle parameters: $(0.5,-0.17;\dfrac{1}{14}),(0.5,-0.5;\dfrac{1}{14})$ and $(0.5,-0.8;\dfrac{1}{14})$. $P_{max}=0.1$ (upper left), $P_{max}=1.0$ (upper right), $P_{max}=10.0$ (lower left) and $P_{max}=40.0$ (lower right) in region $V^0$.

**Figure 6:**
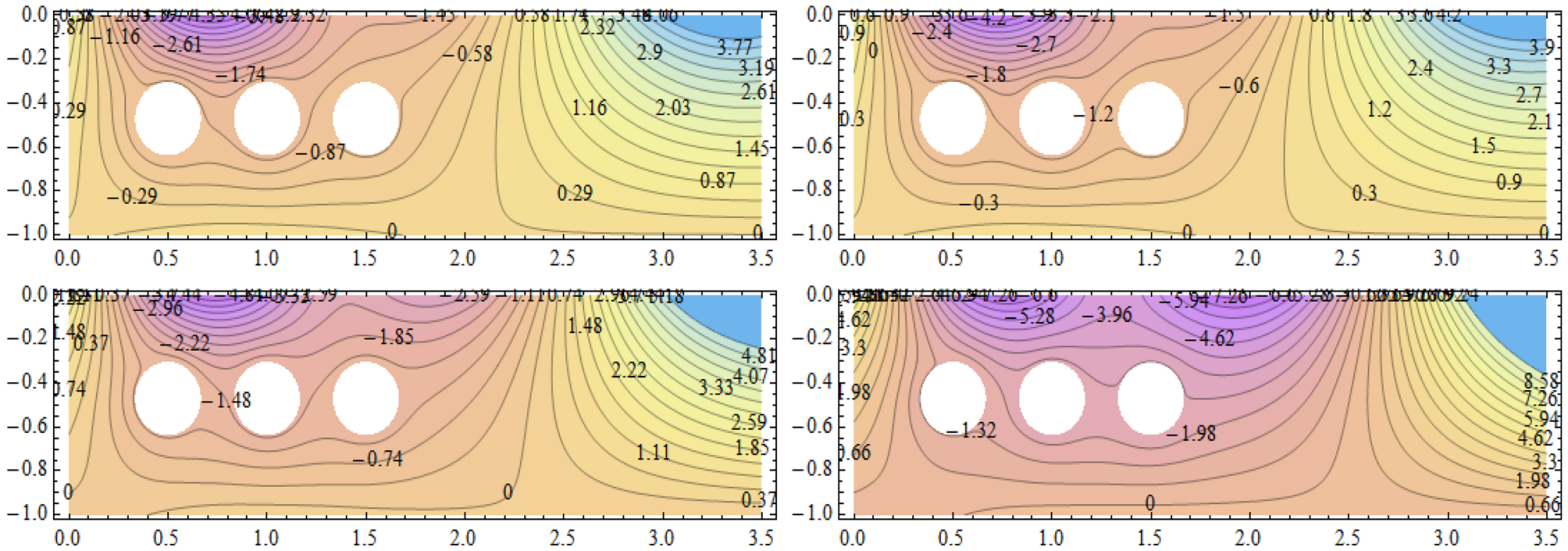
Streamfunction and level curves for three circular obstacles in horizontal setting. Obstacle parameters: $(0.5,-0.47;\dfrac{1}{12}),(1.5,-0.47;\dfrac{1}{12})$ and $(1,-0.47;\dfrac{1}{12})$. $P_{max}=0.1$ (upper left), $P_{max}=1.0$ (upper right), $P_{max}=10.0$ (lower left) and $P_{max}=40.0$ (lower right) in region $V^0$.

**Figure 7:**
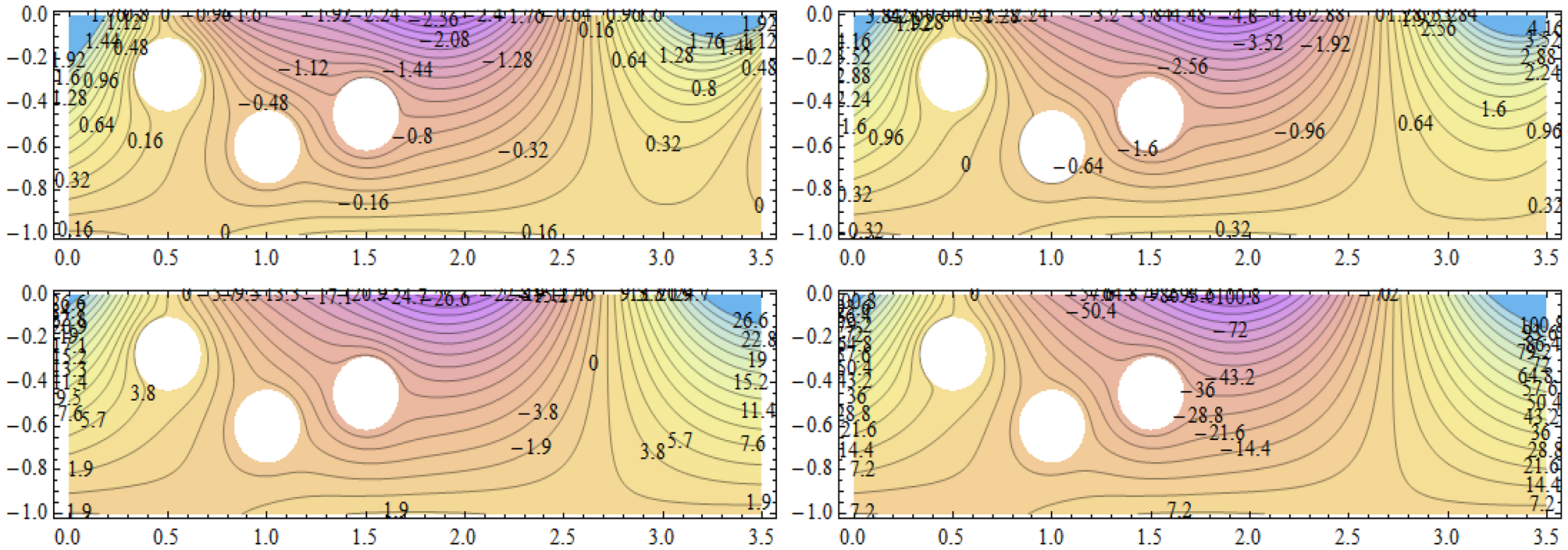
Streamfunction and level curves for three circular obstacles at different depths. Obstacle parameters: $(0.5,-0.27;\dfrac{1}{12}),(1.5,-0.45;\dfrac{1}{12})$ and $(1,-0.6;\dfrac{1}{12})$. $P_{max}=0.1$ (upper left), $P_{max}=1.0$ (upper right), $P_{max}=10.0$ (lower left) and $P_{max}=40.0$ (lower right) in region $V^0$.

**Figure 8:**
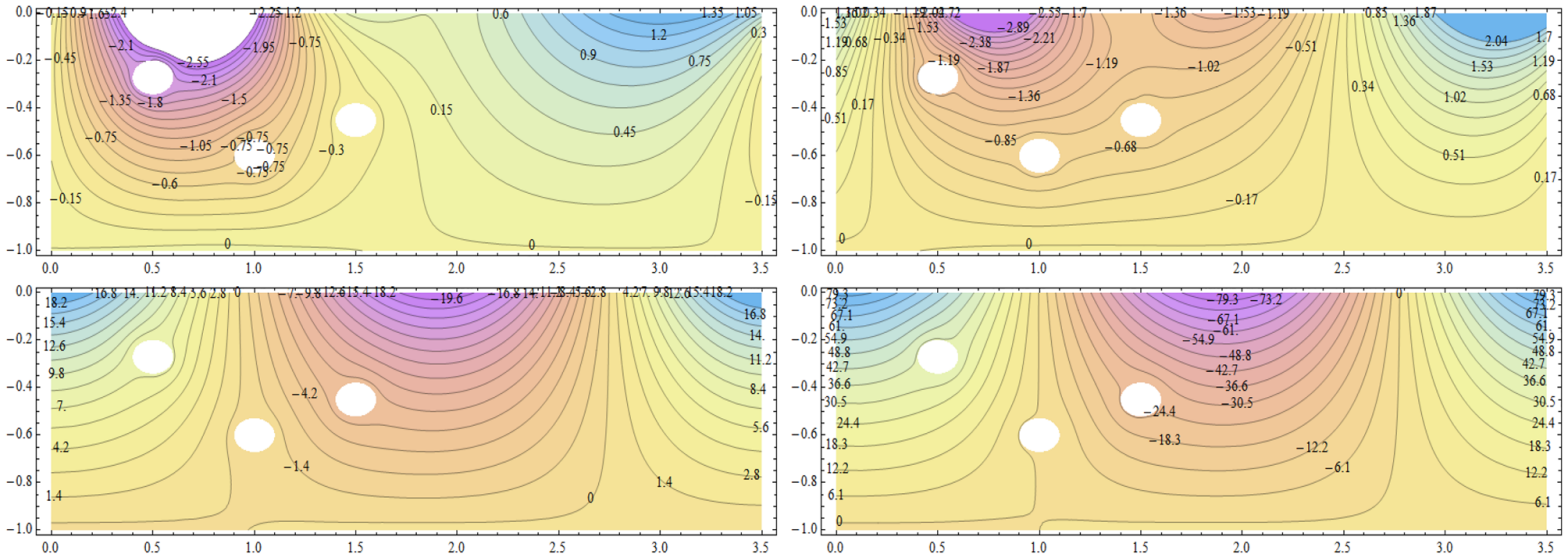
Streamfunction and level curves for three elliptical obstacles at different depths. Obstacle parameters: $(0.5,-0.27,0.05,0.036),(1.5,-0.45,0.05,0.036)$ and $(1,-0.6,0.05,0.036)$. $P_{max}=0.1$ (upper left), $P_{max}=1.0$ (upper right), $P_{max}=10.0$ (lower left) and $P_{max}=40.0$ (lower right) in region $V^0$.

1. **Parabolic surface pressure profile in the absence of obstacles** We have considered a numerical application with parameters:
  $$\begin{aligned} \kappa = 1.5 \end{aligned}$$
  which lies well within the region of validity of the linear theory provided the height of the free surface above the equilibrium position is small enough compared to the water depth. The surface pressure excess distribution was taken of parabolic profile with support [0, *a*] and maximum height $P_{max}$. This is shown in Fig. 2 for $P_{max}=1$ and $a=3.87$. The influence of this maximum pressure on the wave amplitudes at infinity is recorded in Table 1. As expected, one notices that an increase of the surface pressure excess is accompanied by an increase of both wave amplitudes at infinity, clearly expressing the process of transmission of energy from the applied pressure system to the fluid. However, the results in the table show that the increase in the coefficient $T_0$ is slower than that of the coefficient $R_0$. Concerning the wave amplitudes $R_0$ and $T_0$ at both infinities, their sum is equal to the amplitude $I_0$ of the incident wave from mass conservation. However, the sum $\vert R_0 \vert ^2 + \vert T_0 \vert ^2$ exceeds $\vert I_0 \vert ^2$ by a quantity arising from the energy transfer from the surface pressure system to the fluid. Figure 3 represents the system of streamlines in region $V^0$ in the absence of obstacles. The flow region in the whole channel is composed of rectangular cells separated by vertical lines with zero value (C.f.(*I*)) as an expression of the harmonicity in time of the considered motion. The absolute values assumed by the stream function on the level curves within each cell increase from a zero value at the bottom line to a maximum value at the free surface. An increase in the value of $P_{max}$ yields an increase in the absolute values on the contour lines of the stream function. This means an increase in the velocities in the flow within this region. Figure 4 is a 3*D* representation of the stream function. It complies with the zero value on the bottom line, as well with the general requirements for harmonic functions. The variations in this function become larger as one moves from the bottom line towards the flow surface.
2. **Parabolic pressure profile and three circular vertical obstacles** For all the following cases, we have taken $a=3.5$, while $12 \le n \le 16$. For all the circular obstacles in Figures, we have noted between parentheses three parameters: The (*x*, *y*)-coordinates of the center of the circle, and the value of the radius. For elliptical obstacles, the parentheses include four parameters: The coordinates of the center of the ellipse, and the semi-lengths of the major and the minor axes. For the vertically disposed obstacles appearing in Fig. 5, the errors in satisfying the condition of constancy of the streanfuntion separately on the surfaces of the obstacles are shown in Table 2.
3. **Parabolic pressure profile and three circular horizontal obstacles** For the horizontally disposed obstacles appearing in Fig. 6, the errors in satisfying the condition of constancy of the streanfuntion separately on the surfaces of the obstacles are shown in Table 3.
4. **Parabolic pressure profile and three circular obstacles at different depths** For the obstacles placed at different depths shown in Fig. 7, the errors in satisfying the condition of constancy of the streanfuntion separately on the surfaces of the obstacles are shown in Table 4.
5. **Parabolic pressure profile and three elliptical obstacles at different depths** For the elliptical obstacles placed at different depths shown in Fig. 8, the errors in satisfying the condition of constancy of the streanfuntion separately on the surfaces of the obstacles are shown in Table 5.

**Table 1:** Effect of maximum surface pressure excess on wave amplitudes at infinity.

$P_{max}$	$\frac{R_0}{I_0}$	$\vert \frac{R_0}{I_0} \vert$	$\frac{T_0}{I_0}$	$\vert \frac{T_0}{I_0} \vert$	$\vert \frac{R_0}{I_0} \vert ^2 + \vert \frac{T_0}{I_0} \vert ^2$
0.1	$0. - 0.00836 i$	0.00836	$1. + 0.00836 i$	1.00003	1.00014
0.5	$0. - 0.04182 i$	0.04182	$1. + 0.04182 i$	1.00087	1.00350
1.0	$0. - 0.08364 i$	0.08364	$1. + 0.08364 i$	1.00349	1.01399
1.5	$0. - 0.12546 i$	0.12546	$1. + 0.12546 i$	1.00784	1.03148
2.0	$0. - 0.16728 i$	0.16728	$1. + 0.16728 i$	1.01389	1.05597

**Table 2:** Errors in satisfying the condition of constancy of the streamfunction separately on the surfaces of three circular obstacles in vertical setting.

$P_{max}$	$E_1$	$E_2$	$E_3$
0.1	0.00203022	0.000939514	0.000869302
1.0	0.00192003	0.000798075	0.00078689
10.0	0.00112968	0.000675697	0.000759486
40.0	0.00337063	0.00534607	0.00279102

**Table 3:** Errors in satisfying the condition of constancy of the streamfunction separately on the surfaces of three circular obstacles in horizontal setting.

$P_{max}$	$E_1$	$E_2$	$E_3$
0.1	0.000558089	0.000342163	0.00100268
1.0	0.000627601	0.000319895	0.000997635
10.0	0.00103995	0.000371011	0.000965736
40.0	0.00255966	0.00149389	0.0011199

**Table 4:** Errors in satisfying the condition of constancy of the streamfunction separately on the surfaces of three circular obstacles placed at different depths.

$P_{max}$	$E_1$	$E_2$	$E_3$
0.1	0.000259692	0.000771524	0.000534421
1.0	0.000505241	0.00122829	0.000931948
10.0	0.00307526	0.00579594	0.00491127
40.0	0.0116804	0.0210215	0.0181773

**Table 5:** Errors in satisfying the condition of constancy of the streamfunction separately on the surfaces of three elliptical obstacles placed at different depths.

$P_{max}$	$E_1$	$E_2$	$E_3$
0.1	0.0000216704	0.00218154	0.001825
1.0	0.00352019	0.00264083	0.00185648
10.0	0.0058002	0.00805739	0.00441614
40.0	0.0259929	0.0388764	0.017372

## Conclusions

A method proposed earlier in^1^ has been modified to treat the problem of multiple scattering and power transfer from an incident harmonic wave and a variable oscillatory surface pressure of finite support of the same harmonic time dependence, to an infinite, homogeneous water layer in the presence of a finite number of submerged obstacles. Such a model simulates the generation and propagation of waves in oceans during climate variations. In addition, it can have important environmental applications in oscillating water columns.

The decomposition of the solution into outgoing waves and local perturbations, and the provided analytical formulae for the reflection and the transmission coefficients allow to get a deeper insight into the flow characteristics, as compared with previous investigations. Unlike earlier work, the presented method allows to separate the travelling waves from the local perturbations. Our formulae may be used to calculate the resulting flow for rather general forms of the surface pressure function and for various obstacle shapes.

A new energy relation has been deduced, which permits further investigation of the interplay between the incident wave, the surface pressure excess and the obstacles immersed in the fluid. In the absence of obstacles, the presented solution turns out to be exact and in closed form.

Different discontinuous pressure functions have been considered, including a constant pressure on a finite support. We show the existence of particular frequencies related to the width of the constant pressure function support, for which no outgoing waves are produced. In such cases, the energy provided by the surface pressure excess is exhausted in the generation of standing wave and local perturbations.

Numerical results for a surface pressure excess of parabolic profile in the absence or presence of obstacles are provided. In the case of no obstacles, an increase of this excess pressure is accompanied by an increase of the wave amplitudes at infinity, clearly expressing the energy transfer from the surface pressure system to the fluid.

The case of three obstacles (circular or elliptic) in different settings, in addition to a parabolic surface excess pressure, was considered. It was noticed that the performance of the method becomes less as the maximum pressure excess becomes larger. This is equally true for compact settings of the obstacles, and for larger radii of the circular obstacles. More precisely, the condition of constancy of the streamfunction at the horizontal bottom is harder to satisfy in such cases.

The proposed method is efficient and the arising errors in satisfying the conditions of constancy of the streamfunction at the bottom topography and at the obstacles are small enough only when the maximum excess surface pressure and the radii of the circular obstacles are not too big. Again, the obstacles should not have a compact setting, and placed not too close to the bottom or to the upper bounding surface in order to get reliable results.

Our formulae are not applicable to obstacles in the form of plates, and loses efficiency for slender bodies.

### Supplementary Information

Supplementary Information.

## Data Availability

All data generated or analysed during this study are included in this published article [and its supplementary information files].

## References

[1] M.S. Abou-Dina, A.F. Ghaleb, Multiple wave scattering by submerged obstacles in an infinite channel of finite depth. I. Streamlines, Eur. J. Mech. B/Fluids 59 37-51 (2016). 10.1038/s41598-024-51512-x PMC10806217 38263224

[2] Abou-Dina MS, Hassan FM (2005). Approximate determination of the transmission and reflection coefficients for water-wave flow over a topography. Appl. Math. Comp..

[3] Stoker JJ (1957). Water Waves.

[4] J.V. Wehausen, E.V. Laitone. Surface Waves, Handbuch der Physik, Vol. 9, eds. S. Flügge and C. Truesdell, Springer Verlag, Berlin (1960).

[5] Miles JW (1962). Transient gravity wave response to an oscillating pressure. J. Fluid Mech..

[6] Srokosz, M. A. The submerged sphere as an absorber of wave power. *J. Fluid Mech.* **95**(4), 717–741. 10.1017/S002211207900166X (1979).

[7] Thomas, J. R. The absorption of wave energy by a three-dimensional submerged duct. *J. Fluid Mech.* **104**, 189–215. 10.1017/S0022112081002887 (1981).

[8] Evans DV (1982). Wave-power absorption by systems of oscillating surface pressure distributions. J. Fluid Mech..

[9] A.J.N.A. Sarmento, A.F. de O. Falcão, Wave generation by an oscillating surface-pressure and its application in wave-energy extraction, J. Fluid Mech.150 467-485. (1985) DOI: https://doi.org/10.1017/S0022112085000234

[10] Abou-Dina MS, Helal MA (1990). The influence of a submerged obstacle on an incident wave in stratified shallow water. Eur. J. Mech. (B).

[11] Abou-Dina MS, Helal MA (1992). The effect of a fixed barrier on an incident progressive wave in shallow water. IL Nuovo Cimento (B).

[12] Abou-Dina MS, Helal MA (1995). The effect of a fixed submerged obstacle on an incident wave in stratified shallow water (Mathematical Aspects). IL Nuovo Cimento (B).

[13] Abou-Dina MS, Helal MA (1998). Reduction for the nonlinear problem of fluid waves to a system of integro-differential equations with an oceanographical application. J. Comp. Appl. Math. (CAM).

[14] Darmon A, Benzaquen M, Raphaël E (2014). Kelvin wake pattern at large Froude numbers. J. Fluid Mech..

[15] M. Benzaquen, A. Darmon, E. Raphaël Wake pattern and wave resistance for anisotropic moving objects, Phys. Fluids 26, 092106 (2014). https://doi.org/10.1063/1.4896257

[16] Y. Li, S.Å. Ellingsen, Multiple resonances of a moving oscillating surface disturbance on a shear current, Proc. 25-th (2015) Int. Ocean and Polar Engng. (ISOPE) Conf., Kona, Big Island, Hawaii, USA, June 21-26 (2015).

[17] Li Y, Ellingsen SÅ (2016). Multiple resonances of a moving, oscillating surface disturbance on a shear current. J. Fluid Mech..

[18] Y. Li, B.K. Smeltzer, S.Å, Ellingsen, Transient wave resistance upon a real shear current, Eur. J. Mech. B/Fluids 73, 180-192 (2019).

[19] Alaidrous AA (2021). Transmission and reflection of water-wave on a floating ship in vast oceans. CMC.

[20] Liu PL-F, Higuera P (2022). Water waves generated by moving atmospheric pressure: theoretical analyses with applications to the 2022 Tonga event. J. Fluid Mech..

[21] K.K. Al Arfaj, M.A. Helal, M.S. Abou-Dina, Reflection and transmission of an incident progressive wave by obstacles in homogeneous shallow water, Inf. Sci. Lett. 12 (4) 1959-1971 (2023). https://digitalcommons.aaru.edu.jo/isl/vol12/iss4/19

