# Supplementary material for: Multiple wave scattering by submerged obstacles in an infinite channel of finite depth with surface pressure excess
Source: Sci Rep. 2024 Jan 23;14:1975. doi: 10.1038/s41598-024-51512-x (PMC10806217; doi:10.1038/s41598-024-51512-x)
Supplement: Supplementary file 1 — Supplementary Information. [file 41598_2024_51512_MOESM1_ESM.pdf]

Appendix A. Some coefficients

$$\alpha_{0k} = \frac{1}{J_0\lambda_0 h}\int_{-h}^{0}\frac{x_k\cosh\lambda_0(y+h)}{(y-y_k)^2+x_k^2}\,dy, \quad \alpha_{pk} = \frac{1}{J_p\lambda_p h}\int_{-h}^{0}\frac{x_k\cos\lambda_p(y+h)}{(y-y_k)^2+x_k^2}\,dy,$$

$$\beta_{0k} = \frac{1}{J_0\lambda_0 h}\int_{-h}^{0}\frac{(a-x_k)\cosh\lambda_0(y+h)}{(y-y_k)^2+(a-x_k)^2}\,dy, \quad \beta_{pk} = \frac{1}{J_p\lambda_p h}\int_{-h}^{0}\frac{(a-x_k)\cos\lambda_p(y+h)}{(y-y_k)^2+(a-x_k)^2}\,dy.$$

and

$$\begin{aligned} J_0 &= \frac{1}{h}\int_{-h}^{0}\cosh^2\lambda_0(\sigma+h)\,d\sigma = \frac{1}{2}\left(1+\frac{\sinh 2\lambda_0 h}{2\lambda_0 h}\right), \\ J_p &= \frac{1}{h}\int_{-h}^{0}\cos^2\lambda_p(\sigma+h)\,d\sigma = \frac{1}{2}\left(1+\frac{\sin 2\lambda_p h}{2\lambda_p h}\right). \end{aligned}$$

$$H_m = \int_0^a\left[-2h+\frac{\omega^2}{g}\left(h^2-\sigma^2\right)\right]\cos\frac{m\pi\sigma}{a}\,d\sigma = -2\kappa\frac{a}{h}(-1)^{m+1}\frac{a^2}{m^2\pi^2},$$

$$J_{mk} = \int_0^{\frac{a}{h}}\frac{\frac{y_k}{h}}{\left(\sigma-\frac{x_k}{h}\right)^2+(\frac{y_k}{h})^2}\cos\frac{m\pi h\sigma}{a}\,d\sigma + \kappa\int_0^{\frac{a}{h}}\ln\sqrt{\left(\sigma-\frac{x_k}{h}\right)^2+(\frac{y_k}{h})^2}\cos\frac{m\pi h\sigma}{a}\,d\sigma$$

$$\begin{aligned} I_{0m} &= \int_{-h}^{0}\cosh\frac{m\pi}{a}(y+h)\cosh\lambda_0(y+h)\,dy \\ &= \frac{h}{1-\frac{m^2\pi^2}{\lambda_0^2a^2}}\left[\frac{1}{\lambda_0 h}\cosh\frac{m\pi h}{a}\sinh\lambda_0 h - \frac{1}{(\lambda_0 h)^2}\frac{m\pi h}{a}\sinh\frac{m\pi h}{a}\cosh\lambda_0 h\right], \end{aligned}$$

$$\begin{aligned} I_{mp} &= \int_{-h}^{0}\cosh\frac{m\pi}{a}(y+h)\cos\lambda_p(y+h)\,dy \\ &= \frac{h}{1+\frac{m^2\pi^2}{\lambda_p^2a^2}}\left[\frac{1}{\lambda_p h}\cosh\frac{m\pi h}{a}\sin\lambda_p h + \frac{1}{(\lambda_p h)^2}\frac{m\pi h}{a}\sinh\frac{m\pi h}{a}\cos\lambda_p h\right], \end{aligned}$$

$$
\begin{aligned}
S_0 &= \frac{1}{h}\int_{-h}^{0} (y+h)^2 \cosh \lambda_0 (y+h)\, dy = h^2 \left[\frac{2+\lambda_0^2 h^2}{(\lambda_0 h)^3} \sinh \lambda_0 h - \frac{2}{(\lambda_0 h)^2} \cosh \lambda_0 h\right] \\
S_k &= \int_{-1}^{0} \ln r_k(0, \frac{\sigma}{h}) \cosh\left[\lambda_0 h(\sigma+1)\right]\, d\sigma \\
V_k &= \int_{-1}^{0} \ln r_k(\frac{a}{h}, \frac{\sigma}{h}) \cosh\left[\lambda_0 h(\sigma+1)\right]\, d\sigma \\
U_p &= \int_{-h}^{0} (y+h)^2 \cos \lambda_p (y+h)\, dy = h^3 \left[-\frac{2-\lambda_p^2 h^2}{(\lambda_p h)^3} \sin \lambda_p h + \frac{2}{(\lambda_p h)^2} \cos \lambda_p h\right] \\
S_{kp} &= h\int_{-1}^{0} \ln r_k(0, \frac{\sigma}{h}) \cos\left[\lambda_p h(\sigma+1)\right]\, d\sigma \\
V_{kp} &= h\int_{-1}^{0} \ln r_k(\frac{a}{h}, \frac{\sigma}{h}) \cos\left[\lambda_p h(\sigma+1)\right]\, d\sigma
\end{aligned}
$$

$$
A_{2N} = \frac{a}{\pi h} \frac{1}{\left(\tanh \lambda_0 h + \lambda_0 h \operatorname{sech}^2 \lambda_0 h\right)} \left(a_0 H^{(1)} + i\omega h^2 \frac{1}{h} P^{(1)} + \sum_{k=1}^{2K} a_k J_k^{(1)}\right).
$$

$$
\begin{aligned}
H^{(1)} &= \lambda_0 \int_0^a \sigma^3 \sin \lambda_0 \sigma\, d\sigma = \frac{4\pi N h^3}{(\lambda_0 h)^3} \left(3 - 2\pi^2 N^2\right), \\
P^{(1)} &= -\lambda_0 h \int_0^{\frac{a}{h}} h\sigma P_0(h\sigma)\, \sin \lambda_0 h\sigma\, d\sigma, \\
J_k^{(1)} &= -\int_0^{\frac{a}{h}} \frac{y_k}{(\sigma - x_k)^2 + y_k^2}\, \sin \lambda_0 \sigma\ d\sigma \\
&- \kappa \int_0^{\frac{a}{h}} \ln \sqrt{(\sigma - x_k)^2 + y_k^2}\, \sin \lambda_0 h\sigma\ d\sigma.
\end{aligned}
$$
